# Factors Affecting Aggressiveness among Young Teenage Girls: A Structural Equation Modeling Approach

**DOI:** 10.3390/ejihpe11040098

**Published:** 2021-10-30

**Authors:** Haleh Heizomi, Mohammad Asghari Jafarabadi, Kamiar Kouzekanani, Hossein Matlabi, Mansour Bayrami, Vijay Kumar Chattu, Hamid Allahverdipour

**Affiliations:** 1 Department of Health Education & Promotion, Tabriz University of Medical Sciences, Tabriz 14711, Iran; hhezomih@yahoo.com (H.H.); hm1349@gmail.com (H.M.); 2 Department of Statistics and Epidemiology, School of Medicine, Zanjan University of Medical Sciences, Zanjan 4513956111, Iran; M.asghari862@gmail.com; 3 Road Traffic Injury Research Center, Tabriz University of Medical Sciences, Tabriz 14711, Iran; 4 College of Education & Human Development, TAMUCC, 6300 Ocean Dr., Unit 5818, FC 223, Corpus Christi, TX 78412-5818, USA; kamiar.kouzekanani@tamucc.edu; 5 Department of Psychology, Faculty of Psychology and Educational Sciences, University of Tabriz, Tabriz 14711, Iran; dr.bayrami@yahoo.com; 6 Division of Occupational Medicine, Department of Medicine, Temerty Faculty of Medicine, University of Toronto, Toronto, ON M5C 2CS, Canada; 7 Department of Public Health, Saveetha Medical College and Hospitals, Saveetha Institute of Medical and Technical Sciences, Saveetha University, Chennai 600077, India; 8 Research Center of Psychiatry and Behavioral Sciences, Tabriz University of Medical Sciences, Tabriz 14711, Iran

**Keywords:** aggressiveness, adolescence, aggressive behaviors, girls, psychological well-being, structural equation modeling, young teens

## Abstract

Adolescence is a period of transition for developmental and social domains that may also be accompanied by behavioral problems. Aggressive behavior may be a mental health concern for young teens and is defined as a behavioral and emotional trait that may be distressing for others. This study aimed to understand the factors associated with aggressiveness among young teenage girls. A cross-sectional study was conducted among a sample of 707 female middle school-aged students using multistage random sampling in Tabriz, Iran. The variables of interest were aggressiveness, general health status, happiness, social acceptance, and feelings of loneliness. Structural equation modeling was employed to analyze the data. Low parental support, low satisfaction with body image, high sense of loneliness, and lower perceived social acceptance were found to be the factors influencing aggressiveness. The current study found that the school environment, home environment, individual and interpersonal factors all play a part in aggressiveness. As a result, the contributing elements must be considered when creating and executing successful interventions to improve this population’s psychological well-being.

## 1. Introduction

Adolescence is a period of change for developmental and social domains [1] that may be accompanied by behavioral problems such as aggressive behaviors [2]. Aggressiveness among adolescents may constitute concerns for school health and adolescent health [3,4], and it is defined as a behavioral and emotional response that may be distressing for others [5].

There is some evidence that the prevalence of psychological disorders [6,7] and behavioral problems [8] among adolescent girls is considerable. Aggressive behaviors among adolescent girls are related to serious consequences [9] in various cultures and countries [10,11,12], and its negative impacts have been reported [11,13]. However, aggressive behavior among women has not received widespread attention or concerns [9]. Some studies reported females could have aggressive behavior similar to that of males [14,15], but it seems that research on aggressiveness among females has been insufficient [16], and further research is warranted [17].

Aggressive behavior is typically observed during adolescence. At the same time, it is one of adolescents’ mental health problems associated with various psychological disorders among the youth [18,19]. Approximately 1 in every 10 children suffers from aggressive behaviors or is wearied by peers [20]. In the United States, approximately 10% of adolescents reported being hit, slapped, or physically hurt by a boyfriend or girlfriend during the prior 12 months [21], and approximately 30% reported experiencing psychologically aggressive behaviors in their lifetime [22]. In 2018, UNESCO estimated that approximately 30% of all students annually experience some type of aggression at school [23]. National data from the 2009 Youth Risk Behavior Survey indicate that among the 9th- to 12th-grade girls, 22.9% had been in a physical fight on school property within the prior 12 months [24]. Evidence from school and community-based national surveys supports aggressive behaviors among female adolescents [25]. Consistent with these findings, the prevalence of aggressiveness among Iranian adolescents was reported to be 27% [26].

The literature shows that students with aggressiveness traits in the school environment are at risk of having academic failure, social maladjustment, and lifelong negative and wrong behaviors [27,28,29]. Additionally, it is reported that aggressive behaviors are related to various negative outcomes in adulthood, including low unemployment, social isolation, various social problems, and impaired physical health [19,30]. Involvement in physical violence also increases adolescent girls’ likelihood of engaging with aggressive peer groups, having antisocial partners, becoming pregnant and giving birth as a teen, and engaging in aggressive parenting practices [31,32]. Furthermore, physical and mental health is adversely affected, resulting in depression, emotional distress, externalizing behaviors, pregnancy, and childbearing during adolescence [31,33]. Similarly, involvement in relationally aggressive behaviors has been linked to greater internalizing problems, binge drinking, and tobacco use among females in particular [34,35]. However, in school environments, the teacher–student relationship may be critical to children’s health outcomes [36]. Additionally, family environment and parental support could play a protective role in developing aggressive behavior [37].

Moreover, it is well known that aggressiveness is multifaceted in nature [38] and noticeable among school-aged adolescents; thus, the identification of contributing factors may have theoretical and clinical implications [39]. Therefore, to find a better understanding with a broader perspective on the various determinants of aggressiveness, this study was conducted to apply the socio-ecological framework (SEF). This framework may advance the health promotion programs, moving from a focus on changes on a behavioral or intrapersonal level to a focus on a broader range of changes in the social and environmental context related to behavior- and health-related issues [40]. In order to improve the health of populations, there is a need to investigate multiple levels of influence [41]. Based on the SEF, health and behavior are the outcomes of interest [42], determined by the factors from personal and interpersonal levels to organizational, social, and political levels [43]. The SEF has widely been used to approach different health problems [44,45]. According to the socio-ecological model [46,47], aggressiveness is a product of the biological, social, cultural, and economic factors present at the individual, interpersonal, community, and social levels as a whole. Thus, it is unequally widespread and is likely higher in a country going through an economic, social transition or in a post-conflict situation [46].

Although it has been demonstrated that aggressiveness in the early stages of life is a predictor of many behavioral disorders in later life, there is little research on the factors that influence aggressiveness among adolescent females in Iran. Thus, with such a vision, this study was conducted to identify the risk and protective factors associated with aggressive behaviors in East Azerbaijan, Iran, using the SEF. Given the above context, our study aimed to investigate the individual, interpersonal, organizational, and community-level factors contributing to aggressiveness among young teenage girls. The following hypotheses were proposed as below:

**Hypothesis** **1.** *The individual, interpersonal, organizational, and community-level factors contribute to aggressiveness among adolescent females*.

**Hypothesis** **2.** *Teacher–student relationships and parental support would influence aggressiveness*.

**Hypothesis** **3.** *Body image satisfaction, loneliness, and social acceptance would influence aggressiveness*.

## 2. Materials and Methods

### 2.1. Participants and Procedure

In this cross-sectional design, 805 young teen girls in middle schools in grades 7–9 and aged 12–14 in Tabriz city participated from November 2018 to February 2019. Multistage random sampling was employed to recruit participants in the study. In the first step, from the five educational districts in Tabriz, two middle schools were selected from each district by using a simple random sampling method. Hence, a total of ten schools were included in the study, and then in each middle school, we invited students to participate in the study. In total, 707 young teens girls participated in the study and completed the written questionnaire (Response Rate = 87.8) anonymously and self-reportedly. Furthermore, the participants who were reluctant to participate in the study were excluded.

Additionally, all of the participants were instructed on how to complete the study’s questionnaire. None was receiving any psychiatric treatment. The Ethics Committee approved this study at Tabriz University of Medical Sciences (Ethics Code: IR.TBZMED.REC.1397.527). Parental consent and children assent were obtained for all and voluntarily agreed to take part in data collection.

### 2.2. Measures

#### 2.2.1. Demographics

The demographic data included age, birth order, number of family members, teacher–student relationship (very low, low, moderate, high, very high), friendship quality (very low, low, moderate, high, very high), smoking status (yes/no), being physically active (yes/no), body image satisfaction (yes/no), having parental support (yes/no), sleeping well or having an adequate sleep (yes/no), good parental relationship (yes/no), parental conflict (yes/no), satisfaction with parent–adolescent relationship (yes/no), and the ability to talk with parents about problems (yes/no).

#### 2.2.2. Aggressiveness

Aggressive behaviors were assessed using the new version of the Buss–Perry Aggression Questionnaire, formerly known as the Hostility Inventory [48]. The questionnaire consists of 29 items, measuring four subscales of physical aggression (9 items), verbal aggressiveness (5 items), anger (7 items), and hostility (8 items), using a 5-point Likert-type scale, from “quite looks like me” to “does not look like me at all”. The two negatively stated items (6 and 9) must be reverse-coded. An example item is “Some of my friends think I am a hothead”. The reliability coefficient for the study’s Persian version of the questionnaire was estimated to be 0.85, indicating that the questionnaire’s internal consistency was high. The theoretical range is from 29 to 145, with the higher score indicating aggressiveness. Based on Zivari-Rahman et al.’s study [49], we considered a score above 80 as a higher aggressiveness trait in this study.

#### 2.2.3. General Health

The Persian version of the General Health Questionnaire (GHQ-28) was used to measure the psychological well-being of the respondents [50]. The instrument includes 28 items (reliability coefficient = 0.94), measuring four (4) subscales, each consisting of seven (7) items, focusing on somatic symptoms, anxiety/insomnia, social dysfunction, and severe depression domains. An example item is “Have you recently been feeling perfectly well and in good health”. All items are scored on a 4-point scale (score 0: “not at all”; score 1: “no more than usual”; score 2: “rather more than usual” and score 3: “much more than usual”), resulting in a 0 to 84 theoretical range, in which the higher score indicates poorer mental health status.

#### 2.2.4. Happiness

Happiness was measured by the Persian version of the Oxford Happiness Questionnaire [51]. The questionnaire consists of 29 items using a 6-point Likert-type scale (strongly disagree to strongly agree). An example item is “I do not feel particularly pleased with the way I am.” Higher scores represent higher levels of happiness. The estimate of the reliability coefficient for the translated version of the questionnaire in this study was 0.88.

#### 2.2.5. Social Acceptance

The 33-item Crown and Marlow scale [52] measured social acceptance with yes/no responses. Scores of 0–8 represent the people who are most likely to be excluded from society but not interested in knowing the reasons for their lack of social acceptance. The score range of 9–19 indicates average social acceptance. A score between 20 and 33 demonstrates a high level of social acceptance. An example item is “Before voting, I thoroughly investigate the qualification of all the candidates”. The reliability coefficient, based on our data, was 0.71.

#### 2.2.6. Loneliness

The feeling of loneliness was measured by the Persian version [53] of the UCLA Loneliness Questionnaire [54], consisting of 20 items (11 negatively stated). The study employed a 4-point Likert-type scaling (1 = never, 4 = often), so the theoretical range is from 20 to 80, with higher scores indicating higher levels of loneliness. An example item is “I feel in tune with the people around me”. The reliability coefficient, based on our data, was 0.85. 

### 2.3. Statistical Analysis

The data were analyzed using the Statistical Package for the Social Sciences (SPSS) ( Armonk, N.Y, USA: IBM Corp, 2012)and STATA 14 (Stata Corp, College Station, TX, USA). Skewness and kurtosis indices were examined to confirm the normality of the data. The level of significance was set, a priori, at 0.05. Descriptive statistics were used to summarize and organize the data. Pearson correlation coefficient was applied to indicate the associations between aggressiveness and study major variables.

To determine the relationship between school environment, family environment, and the individual factors and interpersonal factors with psychological factors, Structural Equation Modeling (SEM) was conducted, utilizing maximum-likelihood estimates [55]. All school and family environment variables, the individual factors, and interpersonal factors with psychological factors (aggressiveness and poor psychological well-being) were combined into a single SEM. An acceptable fit was confirmed if (1) root mean square errors of approximation (RMSEA) < 0.08, (2) comparative fit index (CFI) and Tucker–Lewis index (TLI) ≥ 0.90, and (3) standardized root mean square residual (SRMR) < 0.05 [56]. We were able to examine a series of regression equations by the SEM. We hypothesized that school and family environments, individual factors, and interpersonal factors are related to poor psychological well-being through aggressiveness.

## 3. Results

A series of Chi-square tests of independence were performed to examine the associations between demographic and selected characteristics of the participants and aggressiveness, which was treated as a binary variable (aggressiveness, *n* = 81; no aggression, *n* = 626). As shown in Table 1, except for the teacher–student relationship, aggressiveness was not related to any demographic characteristics. Those with non-aggressiveness reported either high (35.80%) or very high (36.10%) relationships with their teachers.

Mean (standard deviation) for aggressiveness was 0.11 (0.31). Mean and standard deviations for the other study variables and their correlation coefficients with aggressiveness are presented in Table 2.

As shown in Table 3, an overwhelming majority of the associations between selected characteristics and aggressiveness were statistically significant. Those with no aggression were non-smokers (90.60%), had adequate sleep (85.90%), were satisfied with their body image (86.60%), had no parental conflict (59.40%), enjoyed parental support (90.40%), reported good relations with their parents (89.60%), were satisfied with parent–adolescent relations (84.80%), and could talk with parents about their problems.

**Figure 1:**
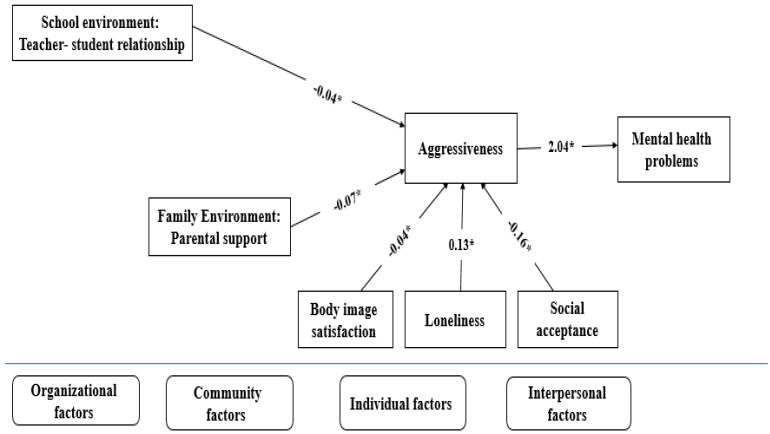
Conceptual model of factors affecting aggressiveness. * *p* < 0.05.

Figure 1 depicts the associations between personal, interpersonal, organizational, and community factors and aggressiveness. The appropriate indices (X2 = 26.64, df = 6, X2/df = 4.44, N = 707, *p* < 0.05, CFI = 0.96, TLI = 0.90, SRMR = 0.02, RSMEA = 0.07, CI: 0.04 to 0.10) showed the model fitted the data. In the final model, the factors influencing aggressiveness were low teacher–student relationship (β = −0.04, *p* < 0.05); low parental support (β = −0.07, *p* < 0.05), low body image satisfaction (β = −0.04, *p* < 0.05), high loneliness (β = 0.13, *p* < 0.05), and low social acceptance (β = −0.16, *p* < 0.05). Teacher–student relationship, parental support, body image satisfaction, and social acceptance were negatively associated with aggressiveness, while the feeling of loneliness showed a positive association with aggressiveness trait. Additionally, aggressiveness was significantly and positively related to poor psychological well-being. The abovementioned factors accounted for 44.30% of the variation.

## 4. Discussion

The present study examined risk and protective factors associated with aggressiveness in a sample of female middle school students in Iran using the SEF. Considering that the SEF has widely been used to approach different health problems [44,45], the topic of aggressiveness is also fairly broad and multifaceted [38]. Nevertheless, in this study, SEF sought, by capturing the necessary risk and protective factors associated with aggressiveness, information peculiar to the ecology of aggressive behaviors among adolescent girls at various levels of influence (Figure 1). For instance, our SEF examined individual-level factors (e.g., body image satisfaction and loneliness), interpersonal factors (e.g., social acceptance) that may increase the risk of aggressiveness, organizational factors (e.g., in the school environment such as a teacher–student relationship), and broad community factors (e.g., in the family environment such as parental support) that provide a broad climate in which one may be aggressive or remain protected. The findings confirmed the mediating role of aggressiveness in elucidating the link between poor psychological well-being and family environment, school environment, individual-level factors, and interpersonal factors. Family, school environments, and individual-level factors (body image satisfaction and loneliness), as well as interpersonal factors (social acceptance), have been consistently linked to aggressiveness-related problems among adolescents in the international scientific literature [38,39]. For children and young teens, the school setting is particularly important in developmental changes and the formation of social behaviors [36,57]. It can help alleviate aggressive characteristics and contribute to the students’ socialization through social and organizational factors [58,59]. We found that the teacher–student relationship, as one of the components of the school environment, was negatively associated with aggressiveness among female adolescents; thus, students who had a better relationship with their teachers demonstrated a lower level of aggressive behavior supported by a previous study [36]. In this regard, it has been demonstrated that classroom teachers play an important role in children’s social, emotional, academic development [60,61] and friendliness relationships [36]. Our findings recommend appropriate communication skills to foster effective interaction between students and teachers to improve mental health and control of aggressive behavior among female adolescents. It seems that in Islamic countries such as Iran, female teenagers mainly interact with their classmates and teachers who are female; thus, having mutual understanding and a good relationship with these female teachers may be helpful to act nonaggressively.

We also found that the family environment can be instrumental in assisting female adolescents in having better psychological well-being and a calm personality. In Islamic societies, young teen girls have limited social activities and interaction with others; thus, family members play an important role in influencing their personality and communication skills. Parental support acts as a protective factor against developing aggressiveness in young teens [37,62]. Additionally, it was reported that living in an intact family lowers the risk of the onset of problem behaviors [58]. In short, research has shown that parents and the family do have a substantial influence on adolescents’ mental health well-being [63]. As a result, improving parent–daughter relationship skills seems to be important.

Body image satisfaction and social acceptance were negatively related to aggressiveness traits among the individual-level and interpersonal factors, respectively, which was also reported in another study [64]. Girls pay more attention to their body image than boys [65]. As a result, we suggest that promoting a healthy lifestyle would help girls to have a satisfying body image and improve calm personality among female adolescents. In the present study, social acceptance was positively associated with better mental health, supported by an earlier study [66]. On the other hand, it has been documented that social acceptance is positively associated with physical activities [67]. However, teenage girls have been identified as a group particularly at risk for physical inactivity in Islamic countries [68,69]. It seems that providing opportunities to engage in physical activity may be an effective way to promote social acceptance, networking, and ultimately improve calmness in young teen girls’ personalities. In the present study, social acceptance was positively associated with better mental health, supported by an earlier study [66].

Another study, however, found a direct and statistically significant relationship between loneliness and aggressive behavior [70]. Thus, it is hypothesized that loneliness can be a reaction to a lack of social relations [71], poor social skills, and lack of social support [72]. The literature suggests that social support is more beneficial to females than males [73]. Therefore, it seems that social support and social engagement may lower aggressive behaviors among female adolescents. We found that aggressiveness leads to poor psychological well-being. The effect of aggressiveness on poor psychological well-being has been well documented [74,75]. In a decade in which the psychological well-being of young people is a public health concern internationally [6,7], it is important to identify and understand the related risk and protective factors of aggressiveness, especially among teens. The study shows that the socioecological model is useful for analyzing youth aggressiveness. It provided evidence to identify the risk and protective factors associated with aggressive behaviors among adolescent females. Socio-cultural theories explain that aggressive behavior is primarily a product of a cultural and social structure in which widespread social inequalities and lack of development opportunities, including unemployment and delinquency, which often present in post-conflict situations and crises, can contribute to creating a subculture of violence in society [65,76]. This study demonstrates that most of the socioecological model’s attributes are relevant explanations of aggressiveness and should be addressed in aggressiveness prevention initiatives.

### Limitations of the Study

Although the present study has several strengths (e.g., data-based, theory-driven, and large sample size), it also has limitations. The first is the self-report nature of data collection, which might have resulted in recall bias. Second, due to the non-experimental nature of the study, no causal inferences were drawn. Third, we did not include male teens in this study so as to investigate gender differences in aggressive behaviors. Aggressiveness may be affected by multiple factors, but numerous factors were affecting our model. Additionally, the relationship between the variables was considered one-way. Still, there may be a reciprocal and two-way relationship between the variables, which we, unfortunately, could not examine in this study. Finally, the investigation was conducted in a single geographic area; thus, the findings may not be generalized to other settings, so replication of the study is recommended. Other factors may influence adolescent girls’ aggressiveness, and we recommend investigating them in future studies.

## 5. Conclusions

The present study, driven by the socio-ecological framework, contributes to individuals’ understanding of aggressiveness among female adolescents, focusing on individual-level, interpersonal, organizational, and broad community factors. It seems that in addition to personal traits, “low parental support” at the community level, “low social acceptance” at the interpersonal level, and a “sense of loneliness” at the individual level can trigger aggressive behaviors among teen females. The evidence shows that if the aggressiveness persists, it will adversely affect psychological well-being and teens’ future relationships. Therefore, identifying effective factors may reduce aggressiveness and ultimately promote the psychological well-being of young teen girls and adolescents.

## Figures and Tables

**Table 1:** Relationships between selected demographic characteristics of participants and aggressiveness.

Variables	Aggressiveness *n* (%)	Non-Aggressiveness *n* (%)	*p*-Value *
Age			
13	20 (8.5)	214 (91.5)	
14	30 (12.9)	202 (87.1)	0.23
15	31 (12.9)	210 (87.1)	
Birth Order			
1	47 (13.1)	313 (86.9)	
2	24 (10.5)	205 (89.5)	0.53
3	7 (9.0)	71 (91.0)	
≥4	3 (7.5)	37 (92.5)	
Number of Family Members			
2	1 (14.3)	6 (85.7)	0.47
3	6 (7.5)	74 (92.5)	
4	51 (12.9)	343 (87.1)	
≥5	23 (10.2)	203 (89.8)	
Teacher–student Relationship			
Very low	8 (9.9)	27 (4.3)	
Low	9 (11.1)	33 (5.3)	<0.05
Moderate	12 (14.8)	116 (18.5)	
High	23 (28.4)	224 (35.8)	
Very high	29 (35.8)	226 (36.1)	
Friend Relationships			
Very low	2 (2.5)	3 (0.5)	
Low	0 (0.0)	11 (1.8)	0.10
Moderate	2 (2.5)	28 (4.5)	
High	22 (27.2)	130 (20.8)	
Very high	55 (67.9)	454 (72.5)	

* Chi-Square Test of Independence.

**Table 2:** Intercorrelations between aggressiveness with major study variables.

Variables	Mean (SD)	X1	X2	X3	X4	X5	X6	X7
X1. Teacher–student relationship	2.91 (1.10)	1						
X2. Parental support	0.88 (031)	0.305 **						
X3. Body image	0.85 (0.35)	0.196 **	0.286 **					
X4. Loneliness	39.79 (10.11)	−0.275 **	−0.339 **	−0.240 **				
X5. Social acceptance	20.35 (4.59)	0.358 **	0.235 **	0.218 **	−0.356 **			
X6. Mental problems	24.71 (16.32)	−0.365 **	−0.402 **	−0.298 **	0.494 **	−0.501 **		
X7. Aggressiveness	0.11 (0.31)	−0.072	−0.140 **	−0.091 *	0.160 **	−0.340 **	0.377 **	1

* *p*-value is significant at *p* < 0.01; ** *p*-value is significant at *p* < 0.001.

**Table 3:** Relationships Between selected characteristics of participants and aggressiveness.

Variables	Aggressiveness *n* (%)	Non-Aggressiveness *n* (%)	*p*-Value *
Smoking status			
Yes	22 (27.2)	59 (9.4)	<0.05
No	59 (72.8)	567 (90.6)	
Being physically active			
Yes	39 (48.1)	325 (51.9)	0.30
No	42 (51.9)	301 (48.1)	
Adequate sleep			
Yes	62 (76.5)	538 (85.9)	<0.05
No	19 (23.5)	88 (14.1)	
Body image satisfaction			
Yes	62 (76.5)	542 (86.6)	<0.05
No	19 (23.5)	84 (13.4)	
Conflict between Parents			
Yes	54 (66.7)	254 (40.6)	<0.05
No	27 (33.3)	372 (59.4)	
Having parental support			
Yes	62 (76.5)	566 (90.4)	<0.05
No	19 (23.5)	60 (9.6)	
Parents good relationship			
Yes	62 (76.5)	561 (89.6)	<0.05
No	19 (23.5)	65 (10.4)	
Satisfaction with parent–adolescent relationship			
Yes	52 (64.2)	531 (84.8)	<0.05
No	29 (35.8)	95 (15.2)	
Talking to your parents about your problems			
Yes	40 (49.4)	433 (69.2)	<0.05
No	41 (50.6)	193 (30.8)	

* Chi-Square Test of Independence.

## Data Availability

The data presented in this study are available on request from the corresponding author (H.A.).

## References

[1] Schlomer G.L., Cleveland H.H., Vandenbergh D.J., Mark E., Feinberg M.E., Neiderhiser J.M., Greenberg M.T., Spoth R., Redmond C. (2015). Developmental Differences in Early Adolescent Aggression: A Gene × Environment × Intervention Analysis. J. Youth Adolesc..

[2] Ye P., Huang Z., Zhou H., Tang Q. (2021). Music-based intervention to reduce aggressive behavior in children and adolescents: A meta-analysis. Medicine.

[3] Haynes P.L., Bootzin R.R., Smith L., Cousins M., Stevens S. (2006). Sleep and aggression in substance- abusing adolescents: Results from an integrative behavioral sleep-treatment pilot program. Sleep.

[4] Crespo-Ramos S., Romero-Abrio A., Martínez-Ferrer B., Musitu G. (2017). Psychosocial variables and overt school violence among adolescents. Psychosoc. Interv..

[5] Kim J.W., Kim H.J. (2007). Relationships among children’s aggression, temperament, home environment, and school adjustment. J. Child. Edu..

[6] Heizomi H., Nadrian H. (2018). What determines psychological well-being among Iranian female adolescents? Perceived stress may overshadow all determinants. Health Promot. Perspect.

[7] Mundy L.K., Simmons J.G., Allen N.B., Viner R.M., Bayer J.K., Olds T., Williams J., Olsson C., Romaniuk H., Mensah F. (2012). Study protocol: The Childhood to Adolescence Transition Study (CATS). BMC Pediatr..

[8] Rabbani A., Mahmoudi-Gharaei J., Mohammadi M., Motlagh M., Mohammad K., Ardalan G., Maftoon F. (2012). Mental health problems of Iranian female adolescents and its association with pubertal development: A nationwide study. Acta Med. Iran..

[9] Straus M.A. (2006). Future research on gender symmetry in physical assaults on partners. Violence Against Women.

[10] Collishaw S., Gardner F., Maughan B., Scot J., Pickles A. (2012). Do historical changes in parent-child relationships explain increases in youth conduct problems?. J. Abnorm. Child Psychol..

[11] Edalati A., Redzuan M. (2010). Women Physical Aggression (A Review). Am. J. Sci..

[12] Fairchild G., Hagan C.C., Walsh N.D., Passamonti L., Calder A.J. (2013). Brain structure abnormalities in adolescent girls with conduct disorder. J. Child Psychol. Psychiatry.

[13] Zahn M., Hawkins S., Chiancone J., Whitworth A. (2008). Girls Study Group: Charting the Way to Delinquency Prevention for Girls.

[14] Obradovic-Tomasevic B., Santric-Milicevic M., Vasic V., Vukovic D., Sipetic-Grujicic S., Bjegovic-Mikanovic V., Terzic-Supic Z., Tomasevic R., Todorovic J., Babic U. (2019). Prevalence and Predictors of Violence Victimization and Violent Behavior among Youths: A Population-Based Study in Serbia. Int. J. Environ. Res. Public Health.

[15] Park S., Chiu W., Won D. (2017). Effects of physical education, extracurricular sports activities, and leisure satisfaction on adolescent aggressive behavior: A latent growth modeling approach. PLoS ONE.

[16] Denson T.F., O’Dean S.M., Blake K.R., Beames J.R. (2018). Aggression in Women: Behavior, Brain and Hormones. Front. Behav. Neurosci..

[17] Rinnewitz L., Parzer P., Koenig J., Bertsch K., Brunner R., Resch F., Kaess M. (2019). A Biobehavioral Validation of the Taylor Aggression Paradigm in Female Adolescents. Sci. Rep..

[18] Bonell C., Allen E., Warren E., Gowan J., Bevilacqua L., Jamal F., Legood R., Viner R.M. (2018). Effects of the Learning Together intervention on bullying and aggression in English secondary schools (INCLUSIVE): A cluster randomised controlled trial. Lancet.

[19] Buchmann A., Hohmann S., Brandeis D., Banaschewski T., Poustka L. (2014). Aggression in children and adolescents. Curr. Top. Behav. Neurosci..

[20] Rub M.A. (2018). An Assessment of Bullying/Victimization Behaviors among Third-Graders in Jordanian Public Schools. Int. J. Res. Educ..

[21] Kann L., Kinchen S., Shanklin S., Flint K.H., Hawkins J., Harris W.A., Lowry R., Olsen E.O. (2014). Youth Risk Behavior Surveillance—United States, 2013. Morb. Mortal. Wkly Rep..

[22] Halpern C.T., Oslak S.G., Mary L., Young M.L., Martin S.L., Kupper L.L. (2001). Partner Violence Among Adolescents in Opposite-Sex Romantic Relationships: Findings from the National Longitudinal Study of Adolescent Health. Am. J. Public Health.

[23] United Nations Educational, Scientific, and Cultural Organization (2018). School Violence and Bullying: Global Status and Trends, Drivers and Consequences.

[24] Eaton D.K., Kann L., Kinchen S., Shanklin S., Ross J., Hawkins J., Harris W.A., Lowry R., McManus T., Chyen D. (2008). Youth risk behavior surveillance-United States, 2007. MMWR Surveill. Summ..

[25] Eaton D.K., Kann L., Kinchen S., Shanklin S., Flint K.H., Hawkins J., Harris W.A., Lowry R., McManus T., Chyen D. (2012). Youth risk behavior surveillance-United States, 2012. MMWR Surveill. Summ..

[26] Ansari H., Kelishadi R., Qorbani M., Mansourian M., Ahadi Z., Motlagh M.E., Ardalan G., Safiri S., Asayesh H., Mohammadi R. (2016). Is Meal Frequency Associated with Mental Distress and Violent Behaviors in Children and Adolescents? the CASPIAN IV Study. Int. J. Pediatr..

[27] Estévez E., Jiménez T.I., Moreno D. (2018). Aggressive behavior in adolescence as a predictor of personal, family, and school adjustment problems. Psicothema.

[28] Salimi N., Karimi-Shahanjarini A., Rezapur-Shahkolai F., Hamzeh B., Roshanaei G., Babamiri M. (2019). Aggression and its predictors among elementary students. J. Inj. Violence Res..

[29] Jenkins L.N., Demaray M.K., Tennant J. (2017). Social, emotional, and cognitive factors associated with bullying. School Psych. Rev..

[30] Odgers C.L., Moffitt T.E., Broadbent J.M., Dickson N., Hancox R.J. (2008). Female and male antisocial trajectories: From childhood origins to adult outcomes. Dev. Psychopathol..

[31] Kirby D., Lepore G. (2007). Sexual Risk and Protective Factors Affecting Teen Sexual Behavior, Pregnancy, Childbearing and Sexually Transmitted Disease: Which Are Important? Which Can You Change?.

[32] Putallaz M., Bierman K.L. (2004). Girls who bully: A developmental and relational perspective. Aggression, Antisocial Behavior, and Violence Among Girls: A Developmental Perspective.

[33] Buka S.L., Stichick T.L., Birdthistle I., Earls F.J. (2001). Youth exposure to violence: Prevalence, risks, and consequences. Am. J. Orthopsychiatry.

[34] Card N.A., Stucky B.D., Sawalani G.M., Little T.D. (2008). Direct and indirect aggression during childhood and adolescence: A meta-analytic review of gender differences, intercorrelations, and relations to maladjustment. Child Dev..

[35] Herrenkohl T.I., Catalano R.F., Hemphill S.A., Toumbourou J.W. (2009). Longitudinal Examination of Physical and Relational Aggression as Precursors to Later Problem Behaviors in Adolescents. Violence Vict..

[36] Sette S., Spinrad T., Baumgartner E. (2013). Links Among Italian Preschoolers’ Socio-Emotional Competence, Teacher-Child Relationship Quality and Peer Acceptance. Early Educ. Dev..

[37] Henneberger A.K., Varga S.M., Moudy A., Tolan P.H. (2016). Family Functioning and High Risk Adolescents’ Aggressive Behavior: Examining Effects by Ethnicity. J. Youth Adolesc..

[38] Xiong R., Xia Y., Li S.D. (2021). Perceived Discrimination and Aggression Among Chinese Migrant Adolescents: A Moderated Mediation Model. Front Psychol..

[39] Kumar M., Bhilwar M., Kapoor R., Sharma P., Parija P. (2016). Prevalence of Aggression among School-Going Adolescents in India: A Review Study. Ind. J. Youth Adol. Health.

[40] Robinson T. (2008). Applying the socio-ecological model to improving fruit and vegetable intake among low-income African Americans. J. Community Health.

[41] Sallis J.F., Owen N., Fisher E. (2015). Ecological models of health behavior. Health Behav..

[42] Glanz K., Rimer B.K., Viswanath K. (2008). Health Behavior and Health Education: Theory, Research, and Practice.

[43] Krug E., Mercy J.A., Dahlberg L.L., Zwi A.B. (2002). The world report on violence and health. Lancet.

[44] Bakhtari F., Jafarabadi M.A., Allahverdipour H., Nikookheslat S.D., Nourizadeh R. (2013). Explaining the role of personal, social and physical environment factors on employed women’s physical activity: A structural equation analysis. Glob. J. Health Sci..

[45] Bakhtari F., Nadrian H., Matlabi H., Sarbakhsh P., Bidar M. (2017). Personal, interpersonal, and organizational predictors of the mode of delivery among urban women: A prospective study with socio-ecological approach. Clin. Nurs. Res..

[46] WHO (2016). Global Plan of Action to Strengthen the Role of the Health System within a National Multisectoral Response to Address Interpersonal Violence, in Particular against Women and Girls, and Against Children.

[47] Dahlberg L.L., Krug E.G., Krug E., Dahlberg L.L., Mercy J.A., Zw A.B., Lozano R. (2002). Violence: A global public health problem. World Report on Violence and Health.

[48] Buss A., Perry M. (1992). The aggression questionnaire. J. Pers. Soc. Psychol..

[49] Zivari-Rahman M., Lesani M., Shokouhi-Moqaddam S. (2012). Comparison of Mental Health, Aggression and Hopefulness between Student Drug-Users and Healthy Students (A Study in Iran). Addict Health.

[50] Moeini B., Shafii F., Hidarnia A., Babaii G.R., Birashk B., Allahverdipour H. (2008). Perceived stress, self-efficacy and its relations to psychological well-being status in Iranian male high school students. Soc. Behav. Pers..

[51] Alipoor A., Noorbala A.A. (1999). A Preliminary Evaluation of the Validity and Reliability of the Oxford Happiness Questionnaire in Students in the Universities of Tehran. Iran J. Psychiatry Clin. Psychol..

[52] Bidaki R., Mousavi S., Bashardoust N., Sabouri Ghannad M., Dashti N. (2016). Individual Factors of Social Acceptance in Patients Infected with Human Immunodeficiency Virus (HIV) at the Yazd Behavioral Consultation Center in Iran. Int. J. High Risk Behav. Addict..

[53] Alamdarlo G., Dehshiri G., Shojaee S., Hakimirad E. (2008). Health and Loneliness Status of the Elderly Living in Nursing Homes Versus Those Living with Their Families. Iran J. Ageing.

[54] Russell D., Peplau L.A., Ferguson M.L. (1978). Developing a measure of loneliness. J. Pers. Assess.

[55] Sodani M., Shogaeyan M., Neysi A. (2012). The effect of group logo-therapy on loneliness in retired men. Res. Cogn. Behav. Sci..

[56] Woo J.P. (2012). The Concepts and Understanding of the Structural Equation Model.

[57] Jessor R. (1993). Successful adolescent development among youth in high-risk settings. Am. Psychol..

[58] Colder C.R., Mott J.A., Flay B.R., Levy S. (2000). The Relation of Perceived Neighborhood Danger to Childhood Aggression: A Test of Mediating Mechanisms. Am. J. Community Psychol..

[59] Howley C., Strange M., Bickel R. (2000). Research about School Size and School Performance in Impoverished Communities: Clearinghouse on Rural Education and Small Schools, Appalachia Educational Laboratory.

[60] Chang L., Liu H., Fung K.Y., Wang Y., Wen Z., Li H., Farver J.M. (2007). The mediating and moderating effects of teacher preference on the relations between students’ social behaviors and peer acceptance. Merril Palmer Q..

[61] Murray C., Murray K.M., Waas G.A. (2008). Child and teacher reports of teacher-student relationships: Concordance of perspectives and associations with school adjustment in urban kindergarten classrooms. J. Appl. Dev. Psychol..

[62] Labella M.H., Masten A.S. (2018). Family influences on the development of aggression and violence. Curr. Opin. Psychol..

[63] Kerestes G., Rezo I., Ajdukovic M. (2019). Links between attachment to parents and internalizing problems in adolescence: The mediating role of adolescents’ personality. Curr. Psychol..

[64] Kartal Yagız A., Kugu N., Semiz M., Kavakci O. (2016). The Relationship Between Anger Expression, Body Image and Eating Attitudes in Social Anxiety Disorder. Turk Psikiyatri. Derg..

[65] Sethi D., Hughes K., Bellis M., Mitis F., Racioppi F. (2014). European Report on Preventing Violence and Knife Crime among Young People.

[66] Twenge J.M., Baumeister R.F., Tice D.M., Stucke T.S. (2002). If you can’t join them, beat them: Effects of social exclusion on aggressive behavior. J. Pers. Soc. Psychol..

[67] Daniels E., Leaper C. (2006). A longitudinal investigation of sport participation, peer acceptance, and self-esteem among adolescent girls and boys. Sex Roles.

[68] Kelishadi R., Qorbani M., Djalalinia S., Sheidaei A., Rezaei F., Arefirad T., Safiri S., Asayesh H., Motlagh M.E. (2017). Physical inactivity and associated factors in Iranian children and adolescents: The Weight Disorders Survey of the CASPIAN-IV study. J. Cardiovasc. Thorac. Res..

[69] Mohebi F., Mohajer B., Yoosefi M., Sheidaei A., Zokaei H., Damerchilu B., Mehregan A., Shahbal N., Rezaee K., Khezrian M. (2019). Physical activity profile of the Iranian population: STEPS survey, 2016. BMC Public Health.

[70] Yavuzer Y., Albayrak G., Kilicarslan S. (2018). Relationships Amongst Aggression, Self-Theory, Loneliness, and Depression in Emerging Adults. Psychol. Rep..

[71] Bhagchandani R.K. (2017). Effect of Loneliness on the Psychological Well-Being of College Students. Int. J. Social. Scienc Humanit..

[72] Rezan A.F. (2007). Psychological well-being predicting loneliness among university students. J. Soc. Sci..

[73] Fiori K.L., Denckla C. (2012). Social support and mental health in middle-aged men and women: A multidimensional approach. J. Aging Health.

[74] Fung A., Gerstein L., Chan Y., Engebretson J. (2015). Relationship of Aggression to Anxiety, Depression, Anger, and Empathy in Hong Kong. J. Child Fam. Stud..

[75] Meyrueix L., Durham G., Miller J., Smalley K.B., Warren J.C. (2015). Association between Depression and Aggression in Rural Women. J. Health Dispar. Res. Pract..

[76] Towsend M.C. (2014). Psychiatric Mental Health Nursing: Concepts of Care in Evidence-Based Practice.

